# Does homeopathic medicine have a preventive effect on respiratory tract infections? A real life observational study

**DOI:** 10.1186/s40248-016-0049-0

**Published:** 2016-03-21

**Authors:** Gianfranco Maria Beghi, Antonio Maria Morselli-Labate

**Affiliations:** Unit of Pulmonary Rehabilitation, Hospital of Casorate Primo, Via Anselmo Dall’Orto, 99, 27022 Casorate Primo (PV), Italy; Department of Medical and Surgical Sciences, Alma Mater Studiorum-University of Bologna, Via Massarenti 9, 40138 Bologna, Italy

**Keywords:** Comparative study, Integrative therapies, Homeopathy, Observational study, Oscillococcinum, Respiratory tract infections

## Abstract

**Background:**

Homeopathic medicine is a branch of integrative medicine that has been gaining increasing popularity. However, its clinical application remains controversial.

To improve the understanding of homeopathy, observational studies-which monitor the effects of homeopathy in real-life clinical settings-are a helpful adjunct to randomized controlled trials.

The goal of this controlled observational study was to investigate the role of the homeopathic medicine in preventing respiratory tract infections (RTIs).

**Methods:**

This retrospective analysis of patients’ medical records focused on a single centre from 2002 to 2011, and examined 459 patients, out of whom 248 were treated with homeopathic medicine (specific extract of duck liver and heart) and 211 were not treated. All patients were followed-up for at least 1 year, and up to a maximum of 10 years.

**Results:**

A significant reduction in the frequency of onset of RTIs was found in both the homeopathic medicine and untreated groups. The reduction in the mean number of RTI episodes during the period of observation vs. the year before inclusion in the study was significantly greater in the homeopathic-treated group than in untreated patients (-4.76 ± 1.45 vs. -3.36 ± 1.30; *p* = 0.001). The beneficial effect of the homeopathic medicine was not significantly related to gender, age, smoking habits or concomitant respiratory diseases when compared to the effect observed in untreated patients.

**Conclusion:**

These results suggest that homeopathic medicine may have a positive effect in preventing RTIs. However, randomized studies are needed before any firm conclusion can be reached.

## Background

Integrative medicine (IM) refers to all those treatments that are not part of conventional healthcare. Homeopathy is a system of IM that was developed in Europe at the end of the eighteenth century employing medicines prepared according to a well-defined procedure starting from mineral, herbal or animal substances. The techniques for preparing these medicines include the repeated dilution of the raw material in hydro-alcoholic solutions or in other excipients, and the ‘succussion’ of the product into different grades [1, 2].

Despite its controversial nature, clinical use of homeopathy has risen steadily in recent years, encouraged by the fact that some of its mechanisms of action have been elucidated and described in randomized controlled trials, meta-analyses or systematic reviews [3–8]. However, other authors have instead negatively evaluated the efficacy of homeopathic medicines, suggesting that they do not yield clinical effects different from placebo [9, 10].

According to the 2012 National Health Interview Survey (NHIS) approximately 5 million adults and 1 million children in the United States used homeopathy in 2011 [11]. According to the 2014 Italian National Institute of Statistics (ISTAT) survey, homeopathic products have been used by approximately 2.5 million people in Italy in the years 2010–2013 and they have been prescribed by over 20,000 physicians [12].

Another debated point in homeopathy is its lack of prescribing standards, as a result of which treatments can be highly individualised for a broad variety of symptoms.

To address these uncertainties, some recent studies have investigated homeopathic treatment protocols using large cohorts of patients and long follow-up periods, so as to monitor the real-world effectiveness of homeopathic medicine in everyday clinical practice [13].

An observational longitudinal study conducted in Italy between 1998 and 2008 analysed the socio-demographic features and the outcomes of a paediatric population treated with homeopathic medicine. The results were promising and indicated a positive therapeutic response, especially in children affected by respiratory diseases [14].

A specific extract of duck liver and heart has been authorized since 1944 and is sold in over 80 countries around the world. It is used for the prevention and treatment of influenza and of viruses that cause influenza-like syndromes. A patented preparation of this homeopathic medicine is commercially available as a Korsakovian dilution (200 K) manufactured from wild duck heart and liver. One dose of this patented preparation contains Anas Barbariae, hepatis and Cordis extractum, as well as the sucrose and lactose as excipients. The ultra-molecular dilutions of this remedy have raised some concerns about its effectiveness, but recently published evidence from in vitro biological models has shown that this kind of homeopathic dilution can elicit significant physiological effects [15, 16]. Thanks to its composition, which may be a reservoir of infective agents responsible of respiratory tract infections (RTIs), this homeopathic medicine is regularly used by many people over the winter months for seasonal colds and airway inflammatory affections [17]. It is well known that RTIs-which comprise any infection of the sinuses, throat, airways or lungs-usually present a viral aetiology. However, RTIs can also be caused by bacteria, and are among the top two causes of morbidity and mortality worldwide [18].

Existing treatment strategies for influenza-like syndromes and RTIs have been shown to have a limited effect on symptoms, also due to the spread of antibiotic and antiviral resistance cases [18, 19]. The aims of those treatments were to enhance the general health of patients by improving symptom control and reducing the frequency of RTI episodes.

This paper presents the results of a retrospective controlled observational study designed to examine health changes, expressed as the reduction in the average number of RTI episodes per year, of a cohort of patients undergoing homeopathic treatment versus a control group of untreated patients, in a real-world setting.

## Methods

### Patients

Between January 1^st^ 2002 and December 31^st^ 2011, overall outcomes were recorded for 459 consecutive patients with RTI or respiratory recurrent infection (IRR) (221 males, 48.1 %; 238 females, 51.9 %. Age range: 1–84 years; mean ± SD: 30.9 ± 25.2 years; IQR: 6–50 years) who were referred to a respiratory diseases specialist office in Agnadello (Cremona, Italy). Inclusion criteria were: all patients included in the study had at least three episodes of RTIs in the year preceding the start of treatment or observation, and at least one year of follow-up after the start of treatment/observation We studied patients with RTI episodes of both upper or lower airways and the presence of a RTI episode was considered when patients presented any ear, throat, airways or lungs infections, as well as sore throat, tonsillitis, laryngitis, sinusitis, stiffy or runny nose, cough, common cold, etc. Subjects who had taken any form of flu vaccine or any other type of vaccine (immunostimulants, bacterial lysates, or similar) were excluded from the observational study. Patients with psychiatric disorders and/or inability to follow the prescriptions were also excluded.

### Ethics

This study was notified to the regional Independent Ethical Committee of Lombardy region. According to the Italian regulation (“Guidelines for the classification of observational study of drug”; May 20^th^, 2008) no approval of the Committee is needed for observational studies. All the subjects gave their written informed consent for the use of their data in this study. The study protocol conforms to the ethical guidelines of the “World Medical Association (WMA) Declaration of Helsinki-Ethical Principles for Medical Research Involving Human Subjects” adopted by the 18^th^ WMA General Assembly, Helsinki, Finland, June 1964.

### First visit (enrollment)

At the first consultation, the current state of health and the nature of each patient’s symptoms were evaluated and recorded in detail. The presence of an RTI was diagnosed by a physical examination, and was defined as comprising one or more of the following complaints: acute inflammation of the throat, red pharyngeal mucosa, ear pain, acute otitis media, streptococcal infection, sinusitis or tonsillitis, inflammation of the larynx. Objective parameters were incorporated into the records whenever possible, together with the patient’s demographic details and clinical diagnosis. For patients with a known pre-existing disease, such as chronic obstructive pulmonary disease (COPD) or bronchial asthma, standard tests (e.g., pulmonary function tests, etc.) were requested.

### Treatment

Out of the 459 enrolled patients, 248 subjects (54.0 %) were treated with a homeopathic medicine (a specific extract of duck liver and heart; Oscillococcinum®, Boiron SA, Messimy, France), while 211 patients (46.0 %) were not treated (control group). The physician initially instructed all 459 patients to take 1 dose of homeopathic medicine (contains 1 g about 200 pillules impregnated with 0.01 mL of the specific extract of duck liver and heart) a week for 8 months (from early September to late April), and to repeat the treatment during the subsequent years. The patients purchased the treatment and took it on their own account. The administration of any kind of vaccine was avoided during the study period for patient choice independently from the medical recommendation.

Adherence to the treatment was evaluated by the medical doctor during subsequent consultation visits and/or by telephone contact. A total of 211 patients were found to be non-compliant (i.e., they did not take the homeopathic medicine as recommended by the medical doctor), and these formed the control group.

The characteristics of the two groups of patients are shown in Table 1. The most represented province in the cohort was Cremona (*n* = 261, 56.9 %), with no significant difference between the treated and untreated group (*p* = 0.553). The distribution of patients according to gender was similar in the two groups (*p* = 0.574) but the patients in the treated group were significantly younger than the patients in the untreated group at the time of inclusion in the study (*p* = 0.032). In fact, the most populated age class was 18–64 years in both groups (*n* = 96, 38.7 and *n* = 105, 49.8 % for the treated and untreated group, respectively) while a total of 67 patients (27.0 %) in the homeopathic medicine group were less than six years old against just 36 such patients in the untreated group (17.1 %), and patients older than 64 were fewer in the treated (*n* = 31, 12.5 %) than in the untreated (*n* = 34, 16.1 %) group.

**Table 1:** Characteristics of the enrolled patients in the treated (specific extract of duck liver and heart) and untreated groups

	Total	Study groups		*p* between groups
		Treated	Untreated	
	(*n* = 459)	(*n* = 248)	(*n* = 211)	
Province of residence:				0.553^b^
- Cremona	261 (56.9 %)	140 (56.5 %)	121 (57.2 %)	
- Bergamo	91 (19.8 %)	48 (19.4 %)	43 (20.4 %)	
- Milan	86 (18.7 %)	51 (20.6 %)	35 (16.6 %)	
- Others	21 (4.6 %)	9 (3.6 %)	12 (5.7 %)	
Gender:				0.574^c^
- Male	221 (48.1 %)	116 (46.8 %)	105 (49.8 %)	
- Female	238 (51.9 %)	132 (53.2 %)	106 (50.2 %)	
Age classes:				0.032^b^
- 1-2 years	29 (6.3 %)	19 (7.7 %)	10 (4.7 %)	
- 3-5 years	74 (16.1 %)	48 (19.4 %)	26 (12.3 %)	
- 6-17 years	90 (19.6 %)	54 (21.8 %)	36 (17.1 %)	
- 18-64 years	201 (43.8 %)	96 (38.7 %)	105 (49.8 %)	
- 65 or more years	65 (14.2 %)	31 (12.5 %)	34 (16.1 %)	
Smoking habits:				0.703^b^
- No smokers	282 (61.4 %)	155 (62.5 %)	127 (60.2 %)	
- Passive smokers	42 (9.2 %)	25 (10.1 %)	17 (8.1 %)	
- Former smokers	62 (13.5 %)	32 (12.9 %)	30 (14.2 %)	
- Current smokers	73 (15.9 %)	36 (14.5 %)	37 (17.5 %)	
No. of concomitant respiratory diseases:				0.168^d^
- None	171 (37.3 %)	86 (34.7 %)	85 (40.3 %)	
- 1 disease	184 (40.1 %)	101 (40.7 %)	83 (39.3 %)	
- 2 diseases	104 (22.7 %)	61 (24.6 %)	43 (20.4 %)	
Concomitant respiratory diseases:				
- COPD^a^	63 (13.7 %)	33 (13.3 %)	30 (14.2 %)	0.787^c^
- Respiratory allergy	195 (42.5 %)	109 (44.0 %)	86 (40.8 %)	0.508^c^
- Asthma	105 (22.9 %)	55 (22.2 %)	50 (23.7 %)	0.739^c^
- Other respiratory diseases	29 (6.3 %)	26 (10.5 %)	3 (1.4 %)	<0.001^c^

^a^ COPD: chronic obstructive pulmonary disease

^b^ Pearson chi-square test

^c^ Fisher’s exact test

^d^ Linear-by-linear chi-square test

One hundred and seventy-seven patients (38.6 %) were smokers (passive, former, or current smokers) and a close relationship was found between smoking habits and both gender (*p* = 0.001) and age (*p* < 0.001). In particular, former smokers were more frequently male than female (*n* = 44, 19.9 vs. n = 18, 7.6 %) while current smokers were more frequently female (*n* = 46, 19.3 vs. *n* = 27, 12.2 %). As far as smoking habits and age were concerned, it should be pointed out that not all patients aged under 18 were non smokers; in fact, there were 3 (10.3), 10 (13.5) and 9 (10.0 %) patients who were passive smokers within the 1–2 year, 3–5 year and 6–17 year age ranges respectively, while one only patient (1.1 %) was a current smoker in the 6–17 year age range. Patients aged 18–64 were mostly nonsmokers (*n* = 99, 49.3 %) while patients over 64 years old were mostly former smokers (*n* = 37, 56.9 %). On the other hand, no significant difference in smoking habits were found between the treated and untreated groups (*p* = 0.703).

A total of 288 patients (62.7 %) had concomitant respiratory diseases. Out of these, 184 (40.1 %) patients had one concomitant respiratory disease and 104 (22.7 %) had two concomitant diseases, so that a total of 392 concomitant respiratory diseases were recorded. Both the number of concomitant respiratory diseases (*p* = 0.168) and the frequencies of chronic obstructive pulmonary disease (COPD), allergies and asthma were not significantly different between the two groups (*p* = 0.787, *p* = 0.508, and *p* = 0.739, respectively), but the frequency of other respiratory diseases, although affecting only a small minority of patients (6.3 %), was significantly higher in the homeopathic^-^treated group (*n* = 26, 10.5 vs. *n* = 3, 1.4 %; *p* < 0.001). COPD was significantly more frequent in males than in females (*n* = 43, 19.5 vs. *n* = 20, 8.4 %; *p* = 0.001), while no significant differences between males and females were found for allergies, asthma and the other respiratory diseases (*p* = 1.000, *p* = 0.150, and *p* = 0.565, respectively). The presence of COPD, allergies and asthma was significantly (*p* ≤ 0.001) related to age and smoking habits. In particular, COPD was highly frequent among the oldest patients (over 64 years old: *n* = 49, 75.4 %) as well as among former smokers (*n* = 42, 67.7 %) and current smokers (*n* = 17, 23.3 %); allergies were more frequently reported by patients aged 6-17 and 18–64 years (*n* = 56, 62.2 and *n* = 99, 49.3 %, respectively) and by passive (*n* = 23, 54.8 %) and nonsmokers (*n* = 132, 46.8 %); finally, asthmatic patients were more frequently present in 18–64 year age span (*n* = 64, 31.8 %) and in passive smokers (*n* = 18, 42.9 %). The presence of other concomitant respiratory diseases was not significantly related to age (*p* = 0.496) or to smoking habits (*p* = 0.679).

### Follow-up

At each subsequent yearly consultation, the patient’s state of health was evaluated and a data form was completed containing the patient’s clinical details and diagnosis. The duration of the observation period varied between 1 and 10 years with a mean ± SD value of 5.3 ± 2.6 years (IQR: 3–7 years). In general, 46 (10.0 %) patients were followed up for 1 year, 150 (32.7 %) for 2–4 years and 263 (57.3 %) for 5 or more years. A total of 21 (4.6 %) patients ended the follow-up before 2012: out of these, 8 (3.2 %) were in the active treatment group and 13 (6.2 %) in the untreated group (*p* = 0.178). The follow-up period was slightly longer for the homeopathic medicine group (5 years or more: *n* = 153, 61.7 vs. *n* = 110, 52.1 %; 2–4 years: *n* = 73, 29.4 vs. *n* = 77, 36.5 %; 1 year: *n* = 22, 8.9 vs. *n* = 24, 11.4 %; homeopathic-treated vs. untreated patients) but the difference did not reach the significance threshold (*p* = 0.055).

### Outcome measures

The primary outcome measure for assessing the effectiveness of the preventive treatment with homeopathic medicine was the reduction in the average number of RTI episodes per year versus the year before inclusion in the study.

A secondary outcome was the evaluation of the possible effects of other variables (gender, age, smoking habits and concomitant respiratory diseases) on the reduction in the average number of RTI episodes.

### Annual numbers of episodes of RTI before inclusion in the study

The mean (±SD) number of episodes of RTI before of inclusion in the study was 5.01 ± 1.66 (range: 3–18; IQR: 4–5). Patients in the group treated with the homeopathic medicine had a significantly higher number of episodes than the untreated group, (5.42 ± 1.69 versus 4.53 ± 1.49; *p* = 0.004). In particular, the majority of patients (*n* = 263, 57.3 %) had at least five episodes of RTI in the year before inclusion in the study: out of these, 184 patients (74.2 %) were in the treated group and 79 (37.4 %) in the untreated group (*p* < 0.001). This finding is in line with the observational type of experimental protocol, since patients were not randomly assigned to one of the two treatment groups, and it reflects everyday clinical medical practice, providing a photograph of how the medicine is actually used.

Gender (*p* = 0.999), smoking habits (*p* = 0.899), and presence of concomitant respiratory diseases (*p* = 0.658, *p* = 0.901, *p* = 0.695, and *p* = 0.830 for COPD, respiratory allergy, asthma and other respiratory diseases, respectively) were not significantly associated with the number of episodes of RTI suffered by patients in the year before the start of the study. Conversely, the number of episodes of RTI before inclusion in the study was inversely related to the age of patients (*p* = 0.001). In particular, children aged 1–2 and 3–5 years had a significantly greater number of episodes (6.03 ± 2.06, *p* = 0.015; 6.41 ± 2.75, *p* < 0.001 respectively) than adult patients (4.45 ± 0.88 episodes in patients aged 18–64 years), while no significant differences were found between both patients aged 6–17 years (4.94 ± 1.32, *p* = 0.206) and older patients (more than 64 years: 4.80 ± 0.85, *p* = 0.693) when compared to adults.

### Statistical analysis

The descriptive analysis of scalar variables was performed using the mean and the standard deviation (SD), as well as the overall and the interquartile (IQR) ranges, whereas categorical variables were described by the absolute and relative frequencies. Fisher’s exact, Pearson’s chi-squared and the linear-by-linear chi-squared tests were used to analyze dichotomous, nominal and ordinal discrete variables respectively, while the general linear models were applied to analyze scalar variables. The z-test was used for checking the skewness of scalar variables: number of RTI episodes reported at each year of follow-up, as well as the mean number of RTI episodes during the follow-up, displayed highly significant positive skewness coefficients (*p* < 0.001) and were transformed before analysis according to the formula log (x + k). On the other hand, the primary outcome measure (i.e., the difference in the average number of RTI episodes per year versus the year before inclusion in the study) showed a highly significant negative skewness (*p* < 0.001), and was accordingly transformed by the exp (x / k) formula. The values of the constant coefficients (k) that maximized the Shapiro-Wilk’s statistics for the normal distribution of each scale variable were chosen in the transformation functions.

The average number of RTI episodes per year and its difference versus the values observed before the study (i.e., the primary endpoint) were assessed by means of eight-way analyses of variance (ANOVA), making adjustments for all eight factors considered in this study (i.e., treatment group, gender, age, smoking habits, and the four concomitant respiratory diseases). Nine-way ANOVA was instead applied to analyse the number of RTI episodes, because the year of follow-up was also considered as an adjusting factor in that analysis. The interactions between treatment group and the other factors were also included in the model to test whether the differences observed between treated and untreated patients differed significantly depending on the classes of the various factors taken into account. To avoid multiple comparisons, simple contrast was used to compare pairs of classes of non-dichotomous discrete variables, while repeated contrast was used to test progressive changes in the number of RTI episodes at each year of follow-up compared to the preceding year. In addition, nested designs were used to test differences between the treatment groups within each class of the various factors, as well as to compare the different classes of the various factors within each treatment group.

Data were managed and analyzed using the IBM SPSS Statistics package (Version 21, IBM, Co., Armonk, NY, USA) and statistical significance was considered to be achieved for two-tailed *p* values lower than 0.05.

## Results

### RTI episodes during the study

**Fig. 1:**
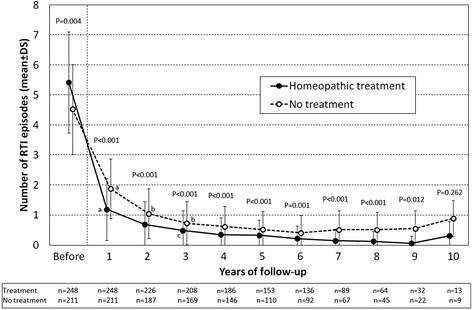
Behaviour of yearly number of RTI episodes before and during the 10-year follow-up in the treated (specific extract of duck liver and heart) and untreated groups. Data are reported as mean ± SD values. The *p* values of the comparison between the two groups are shown in the figure. ^a^ *p* < 0.001, ^b^ *p* = 0.002, ^c^ *p* = 0.006: comparison vs. the previous year within each treatment group

Four thousand and ten episodes of RTI were recorded during the 10–year study period, with a mean number of yearly episodes of 1.40 ± 1.89 (range: 0–18; IQR: 0–2). In particular 44 (9.6 %) patients had no RTI episodes during the follow-up: 42 (16.9 %) in the group taking the specific extract of duck liver and heart and 2 (0.9 %) in the untreated group (*p* < 0.001). The distribution of the average number of episodes of RTI in the 10 years of follow-up is shown in Fig. 1. A significant reduction (*p* < 0.001) in the number of RTI episodes compared to those observed before treatment was found in both groups over the course of the full 10 years of follow-up, showing a progressive decrease of RTI episodes in both groups. In fact, the difference between the number of RTI episode observed at each time compared to the number of RTI episodes observed in the preceding year reached significance from the 1^st^ to the 3^rd^ year for both groups, although the reduction between the 1^st^ and 2^nd^ year did not reach significance in the homeopathic medicine group (*p* = 0.106). The comparison between the two groups showed that, although the treated group had a significantly higher number of RTI episodes in the year before the study (*p* = 0.004), in the first 9 years of follow-up the number of RTI episodes was significantly lower in patients treated with the homeopathic medicine than in the untreated patients while it did not reach significance only in the last year (*p* = 0.262).

The mean number of RTI episodes per year reported by the 459 patients ranged from 0 to 5 with a mean value of 0.90 ± 0.82 (IQR: 0.33–1.20). The treated group had a significantly (*p* < 0.001) lower mean number of RTI episodes per year (0.67 ± 0.67) than patients in the untreated group (1.17 ± 0.89).

### Primary outcome measure: reduction in the number of RTI episodes during follow-up

The change in the mean number of episodes of RTI during the follow-up versus the pre-treatment value ranged from -16.0 to -1.00 (mean ± SD: -4.11 ± 1.55; IQR: -4.83 to -3.30) and was significantly greater (*p* = 0.001) in the treated patients (-4.76 ± 1.45) than in untreated patients (-3.36 ± 1.30).

#### Reduction in the number of RTI episodes according to gender

The reduction in the number of episodes of RTI per year during the follow-up period versus the number of episodes of RTI before inclusion in the study was not significantly related to gender in the overall population (*p* = 0.688), nor within the two groups of patients (homeopathic medicine group: *p* = 0.643; untreated group: *p* = 0.342). This reduction was significantly greater in patients belonging to the treated group when compared to untreated patients of both sexes (males: -4.82 ± 1.31 vs. -3.38 ± 1.67, *p* < 0.001; females: -4.70 ± 1.57 vs. -3.33 ± 0.76, *p* = 0.002), thus indicating that the positive effect of the specific extract of duck liver and heart is similar for both males and females (*p* = 0.309 interaction between gender and treatment).

#### Reduction in the number of RTI episodes according to age

Compared to patients of the untreated group in the same age class, patients in the homeopathic medicine-treated group had a significantly greater reduction in the mean number of RTI episodes per year during the study, as shown in Table 2 (*p* ≤ 0.020). The reduction in the number of RTI episodes differed significantly among age classes in the general population (*p* = 0.010); this was due to the differences in reduction of the number of RTI episodes among age classes in the homeopathic medicine group (*p* = 0.048), whereas no significant differences among age classes were found in the untreated patients (*p* = 0.191). In particular, the effect of the specific extract of duck liver and heart was significantly higher in children aged 3–5 years (*p* = 0.010) than in the adult population (18–64 years). It should be pointed out that the reduction of RTI episodes in both the youngest (1–2 years) and oldest (more than 64 years) patients was similar to the reduction observed in adults (18–64 years). Finally, the comparison of the reductions in RTI episodes observed in homeopathic^-^treated versus untreated patients was not significantly related to age (*p* = 0.754; interaction between age and treatment).

**Table 2:** Difference between the mean number of RTI episodes per year during the follow-up period and the number of episodes of RTI before inclusion in the study stratified according to age classes in the treated (specific extract of duck liver and heart) and untreated groups

Age classes	Total	Study groups		*p* between groups
		Treated	Untreated	
	(*n* = 459)	(*n* = 248)	(*n* = 211)	
1–2 years	-4.22 ± 2.03	-4.77 ± 2.13	-3.17 ± 1.35	0.020
	*p* = 0.361	*p* = 0.746	*p* = 0.366	
3–5 years	-5.01 ± 2.57	-5.47 ± 2.15	-4.16 ± 3.07	0.007
	*p* = 0.005	*p* = 0.010	*p* = 0.138	
6–17 years	-4.32 ± 1.30	-4.98 ± 1.24	-3.33 ± 0.54	0.003
	*p* = 0.083	*p* = 0.063	*p* = 0.526	
18–64 years	-3.83 ± 0.96	-4.44 ± 0.83	-3.28 ± 0.69	0.005
	Reference	Reference	Reference	
65 years and over	-3.63 ± 1.04	-4.24 ± 0.96	-3.07 ± 0.76	<0.001
	*p* = 0.431	*p* = 0.775	*p* = 0.191	
Overall *p* value among age classes	*p* = 0.010	*p* = 0.048	*p* = 0.191	-

The effects observed in the various age classes are also compared with those observed in the adult population (18-64 years)

#### Reduction in the number of RTI episodes according to smoking habits

Similarly to the above findings for gender and age, the homeopathic medicine group showed a significantly greater reduction in the number of RTI episodes compared to untreated patients also within all subgroups of patients with different smoking habits (*p* ≤ 0.011) (Table 3). No significant differences among different smoking classes were found in the overall population (*p* = 0.899), nor within the two groups of treatment (treated group: *p* = 0.684; untreated group: *p* = 0.895), and no significant interaction was found between gender and treatment (*p* = 0.685).

**Table 3:** Difference between the mean number of RTI episodes per year during the follow-up period and the number of episodes of RTI before inclusion in the study stratified according to smoking habits in the treated (specific extract of duck liver and heart) and untreated groups

Smoking habits	Total	Study groups		*p* between groups
		Treated	Untreated	
	(*n* = 459)	(*n* = 248)	(*n* = 211)	
Nonsmokers	-4.25 ± 1.75	-4.92 ± 1.63	-3.43 ± 1.54	0.001
	Reference	Reference	Reference	
Secondhand smokers	-4.16 ± 1.36	-4.69 ± 1.31	-3.38 ± 1.06	0.010
	*p* = 0.749	*p* = 0.645	*p* = 0.973	
Former smokers	-3.72 ± 0.99	-4.21 ± 0.91	-3.20 ± 0.79	0.011
	*p* = 0.680	*p* = 0.920	*p* = 0.492	
Current smokers	-3.90 ± 1.05	-4.59 ± 0.90	-3.23 ± 0.69	<0.001
	*p* = 0.544	*p* = 0.365	*p* = 0.936	
Overall *p* value among smoking habit classes	*p* = 0.899	*p* = 0.684	*p* = 0.895	-

The effects observed in the various smoking classes are also compared with those observed in nonsmokers

#### Reduction in the number of RTI episodes depending on concomitant respiratory diseases

As shown in Table 4, the difference between the mean number of RTI episodes per year during the period of preventive treatment and the mean number of episodes of RTI per year before the inclusion in the study was significantly greater for patients belonging to the homeopathic medicine group than for patients in the untreated group, irrespective of whether they had any of the four types of respiratory diseases (*p* ≤ 0.033). No significant differences of the change in the mean number of RTI episodes per year were found between patients with and without concomitant diseases, both within the general population (*p* ≥ 0.187) and within the two groups of patients (treated group: *p* ≥ 0.152; untreated group: *p* ≥ 0.051). Similarly, the increased reduction observed in the homeopathic medicine group as compared to untreated patients was also unrelated to the presence of any of the four concomitant respiratory diseases (interactions: *p* = 0.380, *p* = 0.120, *p* = 0.051, and *p* = 0.384 for COPD, allergies, asthma and other diseases, respectively).

**Table 4:** Difference between the mean number of RTI episodes per year during the follow-up period and the number of episodes of RTI before inclusion in the study stratified according to the presence of concomitant respiratory diseases in the treated (specific extract of duck liver and heart) and untreated groups

Concomitant respiratory diseases	Total	Study groups		*p* between groups
		Treated	Untreated	
	(*n* = 459)	(*n* = 248)	(*n* = 211)	
COPD^a^				
- No	-4.19 ± 1.60	-4.85 ± 1.49	-3.40 ± 1.36	*p* < 0.001
- Yes	-3.66 ± 1.00	-4.16 ± 0.93	-3.11 ± 0.77	*p* = 0.033
	*p* = 0.330	*p* = 0.152	*p* = 0.949	
Respiratory allergy:				
- No	-4.10 ± 1.63	-4.75 ± 1.63	-3.38 ± 1.29	*p* = 0.003
- Yes	-4.13 ± 1.43	-4.76 ± 1.19	-3.33 ± 1.31	*p* < 0.001
	*p* = 0.187	*p* = 0.860	*p* = 0.051	
Asthma:				
- No	-4.15 ± 1.60	-4.84 ± 1.56	-3.32 ± 1.22	*p* < 0.001
- Yes	-4.00 ± 1.33	-4.47 ± 0.92	-3.84 ± 1.52	*p* = 0.013
	*p* = 0.909	*p* = 0.182	*p* = 0.154	
Other respiratory diseases:				
- No	-4.07 ± 1.45	-4.73 ± 1.26	-3.36 ± 1.30	*p* < 0.001
- Yes	-4.77 ± 2.52	-4.98 ± 2.57	-2.96 ± 0.86	*p* = 0.015
	*p* = 0.394	*p* = 0.980	*p* = 0.360	

^a^ *COPD* chronic obstructive pulmonary disease

## Discussion

The main focus of this retrospective observational study was to explore the effect of the specific extract of duck liver and heart in the prevention of RTIs, by comparing two groups of patients, one that took the medicine and the other that did not.

The results indicate that the evaluated homeopathic drug has a preventive effect on the onset of RTI episodes. Although the role of this drug has been vividly debated, the protective effect observed here is consistent with other studies that have documented its effect on the treatment of flu and flu-like symptoms [9, 20–22]. Thus, our findings add to the growing body of knowledge that could ultimately indicate this homeopathic medicine as a beneficial preventive treatment of RTIs [6].

Moreover, observational data from real-life homeopathic practice are becoming increasingly useful to evaluate the role of this type of IM in treating a wide range of chronic and acute diseases. These results should not be underestimated, also because any gain in health offered by homeopathic medicine, alone or in combination with standard medicine, could be considered of value to the healthcare system in managing the yearly seasonal epidemics that cause RTIs each winter.

An analysis of recent data (2014) of winter all-cause mortality collected by 14 EU countries showed excess mortality levels higher than those seen in the past three winters, especially among older people; these deaths were likely related to RTI [23].

With respect to the analysis of the cohort baseline characteristics, our results indicate that the patients included in the treated group were younger and had more episodes of RTIs during the year before inclusion in the study compared to subjects in the untreated group, whereas the province of residence, gender, smoking habits and distribution of respiratory pathologies were similar for the two groups.

During the observation period, patients treated with the homeopathic medicine had a lower number of respiratory tract infection episodes than untreated patients. In particular, we found that untreated patients who had a mean of 4–5 episodes of exacerbation the year before the study maintained a mean of 2 or less episodes of exacerbation during a subsequent 10-year follow-up without any preventive treatment. It should be pointed out that the values of 4–5 episodes of exacerbation were observed when the patients did not attend the medical consulting while during the study the observed patients had various concomitant pathologies and concomitant pharmacological treatments which were adjusted by the practitioner - for each individual clinical case of both groups - as a function of the patient’s short and long-term medical history. Thus the reduction in the number of infections also among patients in the control group can be accounted for by various factors such as the optimisation of the background therapy. In fact, often, allergic subjects were not following any kind of therapy or asthmatics were not following a bronchodilator background therapy before entering in the study.

The reduction in the mean number of RTI episodes during the observation period versus the mean number of RTI episodes in the year before inclusion in the study was statistically higher in the group of patients treated with the homeopathic medicine than in the untreated group. Both these observations indicate a possible preventive effect of the homeopathic medicine on the onset and development of RTIs.

Importantly, the greater reduction in RTI episodes for the treated group during the overall treatment, compared to patients belonging to the untreated group, was evident irrespective of gender, smoking habits, or the profile of concomitant respiratory diseases. However, the situation for age class distribution was different. Patients treated with specific extract of duck liver and heart did have a greater reduction in the average infectious episodes during the study compared to the year before inclusion than did patients of the untreated group, independently of what age class they belonged to. However, age may have nevertheless played a role in reducing RTI episodes among the treated patients, since the reduction in episodes among these patients seemed greater for patients less than 18 years old (particularly in the 3-5 year age class). This observation is consistent with the pathophysiology of RTIs which are much more prevalent in paediatric/adolescent age than in adults. The exact mechanisms responsible for these observed effects are not clear yet. The literature suggests that ultra-molecular homeopathic dilutions (such as specific extract of duck liver and heart) may exert their effects through a peculiar water structure. However, more evidence of this is needed [24, 25].

This study has several limitations. One particular issue is its observational design which, as documented elsewhere [26], is susceptible to inherent biases associated with the collection of non-random data. In addition, the use of an untreated control group, rather than a placebo-treated one, might be questionable. In this connection, it should be noted that a Cochrane meta-analysis on this same medicine [27] could not give a robust conclusion on the effectiveness of Oscillococcinum owing to the quality of the analysed studies; however, from the report it emerged that those studies had found this homeopathic medicine to have a therapeutic effect for the treatment of influenza-like illness that was different from placebo. The present study shows a snapshot of the use and therapeutic potential of this homeopathic medicine in a real-world, and the results we have found need to be confirmed by large randomized controlled studies. As regards the preventive effect of this medicine, the cumulative result of the meta-analysis was protective but did not reach the statistically significant limit (overall risk ratio (RR) = 0.48; 95 % CI = 0.17–1.34). It should be considered that only two studies were available for the meta-analysis [28, 29], and that in both the RR was found to be protective. In particular, the first study [28] reported a very low RR value of 0.15 (indicating 85 % reduction in the risk of getting sick) although the statistically significant limit was not reached; most likely, this can be attributed to the low number of events (*n* = 50 patients in each group) which “enlarged” the confidence limits. The RR was also protective in the second study (which evaluated 110 Oscillococcinum-treated and 117 placebo-treated patients) [29] and proved statistically significant, although it was less noticeable than the RR of the first study (RR = 0.62; thus a risk reduction of 38 %). Conversely, the main strength of this study is that it provides objective data on the effects of homeopathic care for a frequent and self-limiting condition, for which the use of antibiotics is often ineffective or even inadvisable [30], in a fairly large number of patients. While there is still not enough robust evidence that homeopathic medicine has an effect on RTIs (thus, further controlled, randomized studies are needed), the potential benefits of homeopathic care in reducing antibiotic consumption has been noted [31, 32]. A nationwide French survey of 518 adults and children with upper respiratory tract infections (URTIs) compared antibiotic and antipyretic/anti-inflammatory drug usage, URTI symptom resolution and occurrence of potentially-associated infections in patients in care with practitioners having different levels of prescribing preferences (i.e., homeopathic, general or mixed practice). The results of this study indicated that patients followed by homeopathic practitioners showed significantly lower consumption of antibiotics and antipyretic/anti-inflammatory drugs with respect to those treated by general or mixed practice practitioners, but still had a similar evolution in related symptoms [30].

Finally, it should be pointed out that the evaluation of any medical intervention should take into account not only therapeutic efficacy, but also other factors such as adverse events, costs and compliance. In this observational study the patients treated with the preventive homeopathic medicine reported no adverse reactions, and none of these patients has ever shown and/or reported discomfort in tolerating this homeopathic medicinal product during the period of observation.

## Conclusions

The efficacy of homeopathy is often called into question, mainly owing to the lack of a robust body of evidence [33]. The respiratory tract infections usually have a strong impact on individual well-being, thus it is understandable why the percentage of patients who use homeopathic medicines worldwide is constantly rising, and patient satisfaction with such remedies is usually high [34].

This study suggests that the treatment with this specific homeopathic medicine could be a valuable tool for preventing respiratory tract infections especially in light of the absence of adverse reactions emerged and considering the good evaluation of the relationship between benefits and risks/costs. Although there are limitations for the conclusions that can be drawn from this observational study, it provides a snapshot of the informational role this medicine can play in everyday clinical practice, and further randomized controlled trials are needed before definitive conclusions can be reached.

## Abbreviations

ANOVA: analysis of variance
COPD: chronic obstructive pulmonary disease
IM: integrative medicine
IQR: interquartile range
RR: risk ratio
RTI: respiratory tract infection
SD: standard deviation
URTI: upper respiratory tract infection

## References

[1] Swayne J (2008). Truth, proof and evidence: homeopathy and the medical paradigm. Homeopathy.

[2] World Health Organization (2009). Safety issues in the preparation of homeopathic medicines.

[3] Reilly DT, Taylor MA, Campbell J, Beattie N, McSharry C, Aitchison T (1994). Is evidence for homoeopathy reproducible?. Lancet.

[4] Taylor MA, Reilly D, Llewellyn-Jones RH, McSharry C, Aitchison TC (2000). Randomised controlled trial of homeopathy versus placebo in perennial allergic rhinitis with overview of four trial series. BMJ.

[5] Jonas WB, Kaptchuk TJ, Linde K (2003). A critical overview of homeopathy. Ann Intern Med.

[6] Mathie RT (2003). The research evidence base for homeopathy: a fresh assessment of the literature. Homeopathy.

[7] Bellavite P, Marzotto M, Chirumbolo S, Conforti A (2011). Advances in homeopathy and immunology: a review of clinical research. Front Biosci.

[8] Cucherat M, HaughMC GM, Boissel JP (2000). Evidence of clinical efficacy of homeopathy. A meta-analysis of clinical trials. Eur J Clin Pharmacol.

[9] Chirumbolo S (2013). Oscillococcinum®: misunderstanding or biased interest?. Eur J Intern Med.

[10] Shang A, Huwiler-Müntener K, Nartey L, Jüni P, Dörig S, Sterne JA (2005). Are the clinical effects of homoeopathy placebo effects? comparative study of placebo-controlled trials of homoeopathy and allopathy. Lancet.

[11] Homeopathy: An Introduction. National Center for Complementary and Integrative Health (NCCIH). https://nccih.nih.gov/health/homeopathy. Accessed date 8 June 2015.

[12] Annuario Statistico Italiano. ISTAT 2014. http://www.istat.it/it/archivio/134686. Accessed date 8 June 2015.

[13] Lindqvist E, Saxne T, Geborek P, Eberhardt K (2002). Ten year outcome in a cohort of patients with early rheumatoid arthritis: health status, disease process, and damage. Ann Rheum Dis.

[14] Rossi E, Bartoli P, Panozzo M, Bianchi A, Da Frè M (2010). Outcome of homeopathic treatment in pediatric patients: an observational study from 1998 to 2008. Europ J Integr Med.

[15] Clausen J, van Wijk R, Albrecht H (2011). Review of the use of high potencies in basic research on homeopathy. Homeopathy.

[16] Chirumbolo S, Brizzi M, Ortolani R, Vella A, Bellavite P (2009). Inhibition of CD203c membrane up-regulation in human basophils by high dilutions of histamine: a controlled replication study. Inflamm Res.

[17] Homeopathic Pharmacopoeia Convention of the United States (2012). Monograph: Anas barbariae hepatis et cordis extractum.

[18] Zumla A, Memish ZA, Maeurer M, Bates M, Mwaba P, Al-Tawfi J (2014). Emerging novel and antimicrobial-resistant respiratory tract infections: new drug development and therapeutic options. Lancet Infect Dis.

[19] Kronman MP, Zhou C, Mangione-Smith R (2014). Bacterial prevalence and antimicrobial prescribing trends for acute respiratory tract infections. Pediatrics.

[20] Casanova P, Gerard R (1988). Results of three years of randomised, multicentre studies on Oscillococcinum/placebo.

[21] Papp R, Schuback G, Beck E, Burkardt G, Bengel J, Lehrl S (1998). Oscillococcinum in patients with influenza-like syndromes: a placebo controlled double-blind evaluation. Br Homeopath J.

[22] Marrari LA, Terzan L, Chaufferin G (2012). Oscillococcinum for influenza treatment. Ann Ist Super Sanita.

[23] European Centre for Disease Prevention and Control (2014). Annual epidemiological report 2014-respiratory tract infections.

[24] Demangeat JL, Gries P, Poitevin B, Droesbeke JJ, Zahaf T, Maton F (2004). Low-field NMR water proton longitudinal relaxation in ultrahighly diluted aqueous solutions of silica lactose prepared in glass material for pharmaceutical use. Appl Magn Reson.

[25] Demangeat JL (2009). NMR water proton relaxation in unheated and heated ultrahigh aqueous dilutions of histamine: evidence for an air-dependent supramolecular organization of water. J Mol Liq.

[26] Concato J, Shah N, Horwitz RI (2000). Randomized, controlled trials, observational studies, and the hierarchy of research designs. NEJM.

[27] Mathie RT, Frye J, Fisher P (2015). Homeopathic Oscillococcinum® for preventing and treating influenza and influenza-like illness. Cochrane Database Syst Rev.

[28] Selkova EP, Semenenko TA, Gorbachev IA (2005). Use of the medicine Oscillococcinum for the prevention and treatment of influenza and acute respiratory viral infection. (A) Infectionni Bolezni.

[29] Selkova EP, Semenenko TA, Gorbachev IA (2005). Use of the medicine 745 Oscillococcinum for the prevention and treatment of influenza and acute 746 respiratory viral infection. (B) Infectionni Bolezni.

[30] Grimaldi-Bensouda L, Bégaud B, Rossignol M, Avouac B, Lert F, Rouillon F (2014). Management of upper respiratory tract infections by different medical practices, including homeopathy, and consumption of antibiotics in primary care: the EPI3 cohort study in France 2007–2008. PLoS ONE.

[31] Kirkby R, Herscu P (2010). Homeopathic trial design in influenza treatment. Homeopathy.

[32] Trichard M, Chaufferin G, Nicoloyannis N (2005). Pharmacoeconomic comparison between homeopathic and antibiotic treatment strategies in recurrent acute rhinopharyngitis in children. Homeopathy.

[33] Linde K, Jonas W (2005). Are the clinical effects of homoeopathy placebo effects?. Lancet.

[34] Van Wassenhoven M, Ives G (2004). An observational study of patients receiving homeopathic treatment. Homeopathy.

